# Safety and Efficacy of Cangrelor Among Three Antiplatelet Regimens During Stent-Assisted Endovascular Treatment of Unruptured Intracranial Aneurysm: A Single-Center Retrospective Study

**DOI:** 10.3389/fneur.2022.727026

**Published:** 2022-03-04

**Authors:** Mourad Cheddad El Aouni, Elsa Magro, Mohamed Abdelrady, Michel Nonent, Jean Christophe Gentric, Julien Ognard

**Affiliations:** ^1^Neuroradiology Unit, Department of Radiology, University Hospital of Brest, Brest, France; ^2^Department of Neurosurgery, University Hospital of Brest, Brest, France; ^3^Laboratory of Medical Information Processing—LaTIM INSERM UMR 1101, Brest, France; ^4^Western Brittany Thrombosis Study Group GETBO EA3878, Brest, France

**Keywords:** aneurysm, unruptured, Cangrelor, antiplatelets, stent-assisted coilling, flow diverter

## Abstract

**Introduction:**

Thromboembolic events represent the most frequent complications of endovascular treatment of unruptured intracranial aneurysm using stent-assisted coilling or flow diverter stents. Dual antiplatelet therapy has become the standard to prevent these but remains unstandardized. We present here a single center experience of 3 standardized antiplatelet regimens during brain aneurysm treatment, while emphasizing the use of the Cangrelor.

**Method:**

We retrospectively reviewed data from patients treated using stent-assisted coilling or flow diverter stents from 2016 to 2021. We collected and compared safety and efficacy data within 6 months of three groups of patients corresponding to three antiplatelet standardized regimens: group T with Ticagrelor, with preprocedural preparation; group E with Eptifibatide, injected during procedure; group C with Cangrelor, injected during procedure.

**Results:**

Data of 112 patients were analyzed and 76 belonged to group T, 21 to group E, and 15 to group C. Eleven events over the 14 recorded were adjudicated to be related to antiplatelets, their repartition did not differ between the 3 groups (*p* = 0.43). All symptomatic events (*N* = 8) were not distributed significantly differently between the 3 groups (*p* = 0.11) and asymptomatic events were also balanced (*p* = 1.00). Of these, 6 subjects had a change in the mRS score at 3–6 months. Thrombo-embolic events represented the most encountered events in the sample: 2 acute ischemic strokes were recorded in group E and 1 in group T; 1 transient ischemic stroke was noted in group E; 4 silent infarcts were found on control MRI (2 belonged to group T, 1 to group E and 1 to group C). Among 3 intracranial bleeding events, 1 was symptomatic in group C, 2 were asymptomatic in group T. On the control evaluation performed at 6 months, there was no significant difference on aneurysmal occlusion (*p* = 0.67).

**Conclusion:**

This single-center retrospective study compared 3 antiplatelet regimens, finding no significant difference in the safety and efficacy in the context of endovascular treatments of unruptured aneurysm using stent or flow diverters. This study adds data for the Cangrelor use and suggests its usefulness in the field of neuro-endovascular intervention. Randomized controlled studies are warranted to confirm these results.

## Introduction

Stent-assisted endovascular therapies of intracranial aneurysms may be complicated by thromboembolic events (1, 2). Dual antiplatelet therapy (DAPT) has become a standard regimen to prevent them (3). Actually, there is no standardized DAPT protocol in interventional neuroradiology, which leads to divergent drugs use (4). The use of aspirin and a P2Y_12_ receptor antagonist is inferred from the cardiology literature (5). Historically, Clopidogrel was the first P2Y_12_ inhibitor to be used. Clopidogrel has the disadvantage of a wide response variability (6) and a duration of effect of more than 5 days (7). Ticagrelor and Prasugrel are faster acting (30 min−4 h) than Clopidogrel [2–8 h (8)] and they also present less interpatient response variability (7). These three P2Y_12_ inhibitors can only be administered orally. According to the cardiology literature (9–11), Eptifibatide has the advantage of being used intravenously, with a short onset (15 min) and offset of action (within 4–8 h) (12). Quite similar in structure to Ticagrelor, Cangrelor also allows intravenous administration (13) and short on/offsets (1 min) with a half-life of 3–5 min. Table 1 summarizes the pharmacokinetic and pharmacodynamic properties of these antiplatelet agents. In 2015, Cangrelor was approved by the U.S. Food and Drug Administration as adjunctive therapy for percutaneous coronary intervention as it was effective in reducing intra-stent thrombosis and the risk of peri-procedural myocardial infarction (14). Table 2 summarizes primary studies that investigated the use of Cangrelor in interventional neuroradiology (15–19). These studies used different doses of Cangrelor either for bolus (15–30 μg/kg) or maintenance (0.75–4 μg/kg/min), furthermore these studies reported various procedures from mechanical thrombectomy to ruptured aneurysm treatment.

**Table 1:** Pharmacokinetic and pharmacodynamic properties of antiplatelet agents.

	**Clopidogrel**	**Prasugrel**	**Ticagrelor**	**Eptifibatide**	**Cangrelor**
Class	Thienopyridine	Thienopyridine	Triazolopyrimidine	GPIIb/IIIa	ATP analog
Administration	Oral	Oral	Oral	Intravenous	Intravenous
Reversibity	Irreversible	Irreversible	Reversible	Irreversible	Reversible
Loading dose	300 mg	60 mg	180 mg	90–180 μg/kg	15–30 μg/kg
Maintenance dose	75 mg (once daily)	10 mg (once daily)	90 mg (twice daily)	0.5–2 μg/kg/min	2–4 μg/kg/min
Onset of effect	2–8 h	30 min−4 h	30 min−4 h	5–15 min	0–2 min
Half-Life	6 h	7 h	8 h	1–3 h	2–5 min
Duration of effect	5–7 days	7–10 days	3–5 days	4–8 h	30–60 min

**Table 2:** Review of the literature on the use of Cangrelor in aneurysm treatments.

**Author**	**Journal**	**Year**	**Unruptured aneurysm (*N*)**	**Ruptured aneurysm (*N*)**	**Use of Cangrelor during intracranial stenting**	**Loading dose**	**Maintenance dose**
Linfante et al.	J NeuroIntervent Surg	2021	1	4	Yes	30 μg/kg	4 μg/kg/min
Godier et al.	British Journal of Anaesthesia	2019	2	5	No	No	0,75 μg/kg/min
Abdennour et al.	Clin Neuroradiol	2020	2	5	Yes	30 μg/kg	4 μg/kg/min
Aguilar et al.	J NeuroIntervent Surg	2019	1	-	Yes	15 μg/kg	2 μg/kg/min
Cortez et al.	Neuroradiology	2021	8	16	Yes	15–30 μg/kg	2–4 μg/kg/min
Cheddad El Aouni et al.	Frontiers in Neurology	2021	15	-	Yes	30 μg/kg	4 μg/kg/min

In this study, we aimed to analyse the clinical safety and efficacy of three standardized antiplatelet regimens used during scheduled endovascular procedures for unruptured aneurysms using stent-assisted coilling (SAC) or flow diverter stents (FD), while emphasizing the place of Cangrelor.

## Method

### Population

Data from all patients treated for intracranial aneurysm from February 2016 to March 2021 at the University Hospital were retrospectively collected. Patients treated by simple coilling and ruptured aneurysms were excluded. Three groups of patients were constituted according to their initial antiplatelet regimen that included Ticagrelor, Eptifibatide or Cangrelor. Patients for which another molecule was used were not considered.

We retrospectively collected basic patient demographics, comorbidity, diagnosis, target aneurysm characteristics and procedure-related outcomes and complications from patient medical records included in a prospectively maintained database of all the interventional neuroradiology procedures performed in the center.

### Procedures

Every indication for endovascular treatment was previously validated during a multidisciplinary meeting. The initial treatment strategy was recorded. According to the center habits, antiplatelet regimen was decided before the procedure. For the treatments with anticipated high risk of bleeding (as turning inside the aneurysm, difficult catheterization of recurrent branches, associated microaneurysms, etc.), intravenous antiplatelets were preferred (without P2Y_12_ loading dose before the procedure). Treatment related details were recorded.

### Anti-platelet Regimen Groups

No platelet aggregation test was performed. All patients received a dose of 75 mg Aspirin 1 day prior to the procedure and on the day of the procedure. Systemic anticoagulation was achieved using an intravenous bolus injection of heparin (50 IU/kg, 3,000–5,000 IU, range) after the femoral/radial artery access followed by an additional 1,000–2,000 IU/h according to the results of the activated clotting time.

Group T used a loading dose of 180 mg Ticagrelor 1 day prior to the procedure, and 90 mg Ticagrelor on the day of the procedure.

Group E used a loading dose of 180 μg/kg Eptifibatide during the procedure. If used, the maintenance dose of Eptifibatide was administered at 2 μg/kg/min, within 2 h.

Group C used a loading dose of 30 μg/kg Cangrelor during the procedure, followed by a maintenance dose at 4 μg/kg/min.

Decision of an oral maintenance therapy of 90 mg Ticagrelor twice a day for group T was made after the procedure as soon as a clinical evaluation was possible. Decision of an oral switching for group E and C was made after the procedure as soon as a clinical evaluation was possible, via a loading dose of an oral P2Y_12_ receptor inhibitor (180 mg Ticagrelor or 60 mg Prasugrel), administered 2 h after discontinuation of Eptifibatide, or 30 min before Cangrelor infusion was stopped. All patients received a daily dose of 75 mg Aspirin as a maintenance therapy after the decision of oral DAPT.

### Safety and Efficacy

Main outcome of interest was the presence of an event directly related to DAPT. Adjudication of such accountability was consensually made (by all investigators) on the standardized WHO-UMC system for case causality assessment by taking into account the events which corresponded to the terms “Certain” and “Probable / Likely” (20), with access to all data.

Other secondary items of safety were recorded mainly as symptomatic/asymptomatic hemorrhage/ischemic or other procedure-related events.

Symptomatic events included: intracranial hemorrhage, ischemic stroke, other neurologic symptoms unrelated to ischemia nor hemorrhage, arterial access site complication requiring surgical management and death from any cause within 3 months. Neurological symptoms were also classified as permanent or transient.

Asymptomatic events included transient ischemic attack and silent ischemia/hemorrhage were assessed on the basis of the control MRI at 3–4 months.

Additional relevant outcomes were shift and description of functional conditions from baseline to 3–6 months using the modified Rankin Scale (mRS) (21).

Endovascular treatment efficacy was passed on the basis of both control catheter angiography and MRI at 3–6 months. Eventual intrastent stenosis was recorded in three categories: unsignificant (<20% of the lumen), moderate (20–39%) and substantial (>40%); and aneurysmal occlusion using the Raymond Roy classification (22). The review of all imaging points were made consensually by the neuro-interventionists, JCG – JO – MA – MCEA, blinded to the clinical data.

### Average Cost of Antiplatelets Regimen

The cost of antiplatelet drugs was reported using the following actual hospital market price: 50 mg Cangrelor powder for concentrate for solution for injection/infusion preparation USD 403.3; 10cc vial (2 mg/ml) Eptifibatide USD 36.87; 100cc vial (0.75 mg/ml) Eptifibatide USD 76.48; 90 mg Ticagrelor USD 1.15; 75 mg Aspegic USD 0.045.

The calculation of the cost was based on the actual consumption of antiplatelet drugs for each patient, extracted from the anesthetic record, taking into account the antiplatelet preparation before the procedure and during the procedure, and without taking into account the cost of the maintenance dose. If an infusion was maintained after the procedure without immediate oral switch, this was also taken into account in the cost. This global cost was then given by the average of this consumption for each regimen group.

### Statistics

Data were analyzed using STATA/MP 16 (Statacorp LP, USA) with the aim to compare the safety and efficacy endpoints between the three groups of antiplatelet regimens. A parsimonious analysis was carried out: when they were more adequate, descriptive statistics were preferred. Quantitative variables were described by median an interquartile range (IQR) and compared using a Kruskal-Wallis test. A Chi-squared or Fisher's exact test was used when comparing frequencies between groups for qualitative variables. A value of *p* < 0.05 was considered significant.

### Ethical Statement

The use of Cangrelor was off-label in this study. In all cases no final alternative was found to intracranial stenting and the choice to treat these patients under Cangrelor was made multidisciplinary. Patients were informed before each treatment of the initial strategy and possible alternatives and gave verbal consent. The study and need for patient informed consent was conducted in accordance with actual laws and ethics and with the Helsinki Declaration and its revisions: as a non-interventional retrospective study, a commitment to compliance (Reference Methodology MR-3) was declared to the French national information science and liberties commission (CNIL), in respect to the General Data Protection Regulation. NCT04504695.

## Results

### Population and Procedure

**Figure 1:**
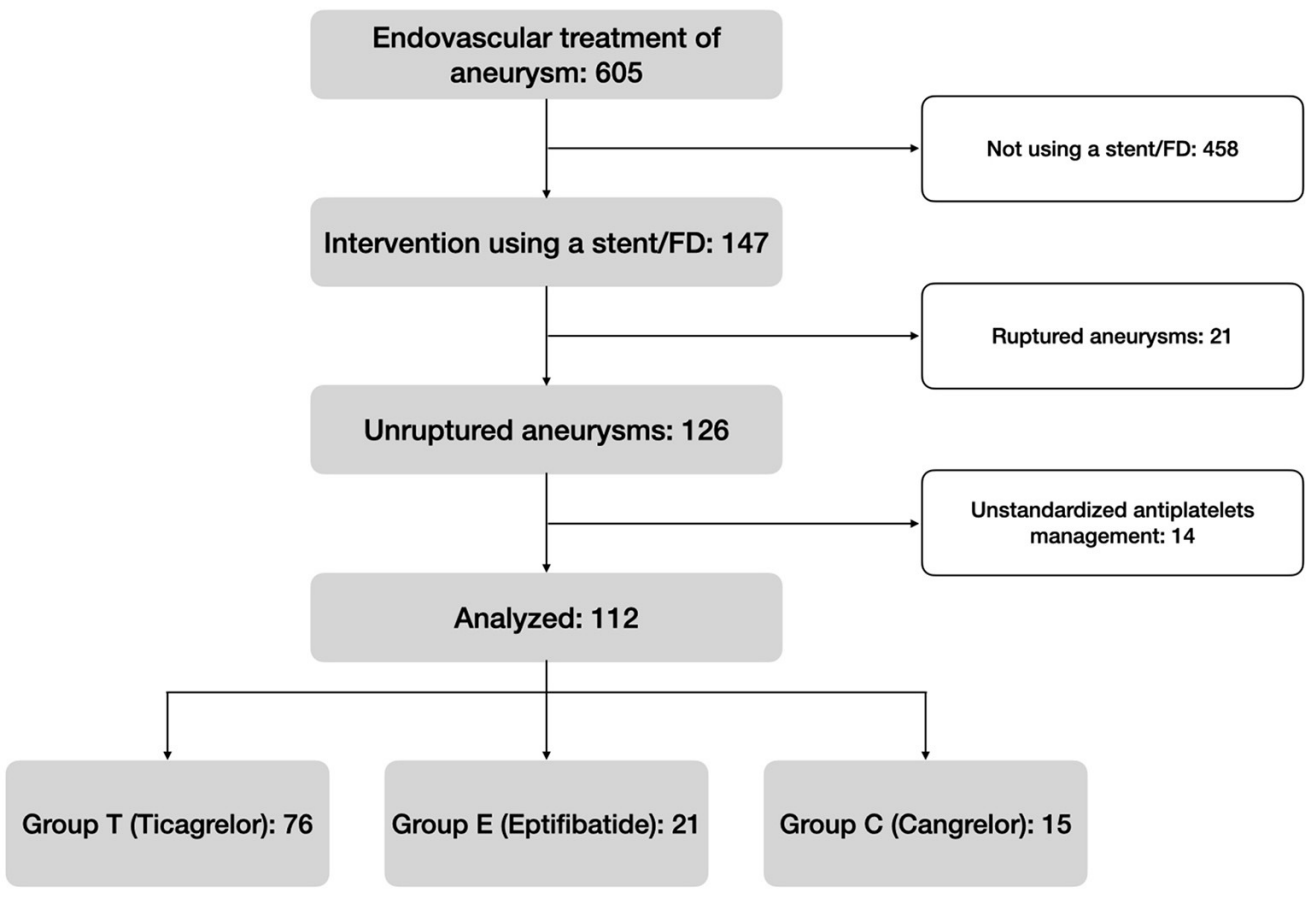
Flowchart of the study.

From February 2016 to March 2021, 605 patients who underwent endovascular intervention for the treatment of an aneurysm were screened. One hundred and forty seven of these were treated by SAC or FD, and 126 were scheduled. Fourteen cases were finally ruled out because the patients did not initially receive neither Ticagrelor, Eptifibatide, nor Cangrelor. Figure 1 summarizes the flow chart.

A total of 112 patients were retrospectively analyzed and 76 belonged to group T (68%), 21 to group E (19%), and 15 to group C (13%). Eighty-one (72%) were women and the median age was 55 years (45–63 years, IQR). No patients had renal failure. Only one stent was used for each procedure. There were no significant differences between the three groups concerning the patients' baseline-characteristics (*p*>0.05), neither regarding aneurysm shape (*p* = 0.494) or size (*p* = 0.970). Table 3 summarizes the general characteristics of the population.

**Table 3:** General characteristics of the population.

**Characteristics**	**Group T**		**Group E**		**Group C**		***p*-value**
Antiplatelet regimen (*n*, %)	76	68%	21	19%	15	13%	
Age in years (median, IQR)	55	46–60	59	43–63	61	45–63	0.559
Gender (female, %)	58	76%	13	62%	10	67%	0.344
Smoking (yes, %)	14	18%	6	29%	4	26%	0.467
Hypertension (yes, %)	21	28%	8	38%	2	13%	0.275
Previous treatment (yes, %)	34	45%	10	48%	9	60%	0.601
Aneurysm type (sacciform, %)	70	92%	21	100%	15	100%	0.494
Aneurysm location							<0.001
Internal carotid artery (*n*, %)	*49*	*64%*	*7*	*33%*	*1*	7%	<0.001
Anterior cerebral artery (*n*, %)	*10*	*14%*	*6*	*29%*	*4*	26%	0.156
Middle ceerebral artery (*n*, %)	*9*	*12%*	*5*	*24%*	*9*	60%	<0.001
Posterior circulation (*n*, %)	*8*	*10%*	*3*	*14%*	*1*	7%	0.804
Size of the aneurysm							0.970
<5 mm (*n*, %)	24	32%	6	29%	5	33%	
5–7 mm (*n*, %)	14	18%	5	24%	4	27%	
7–15 mm (*n*, %)	24	32%	6	29%	3	20%	
>15 mm (*n*, %)	14	18%	4	18%	3	20%	
Treatment modality							<0.001
Flow diverter stent (*n*, %)	59	78%	5	24%	5	33%	<0.001
Laser cut stent (*n*, %)	9	12%	10	48%	1	7%	0.001
Braided stent (*n*, %)	8	10%	6	28%	9	60%	<0.001
Assisted coiling (*n*, %)	28	37%	16	76%	13	87%	<0.001
Unplanned stenting (*n*, %)	0	0%	5	24%	1	7%	

*n: number; %: percentage*.

Half (*N* = 57) of the intervention consisted in SAC and there were significantly less SAC in group T (*p* < 0.001; 37 vs. 76% in group E and 87% in group C). Seven stent placements (6%) were not initially planned. Considering the type of stents used, 20 (18%) were open-cell stents (Neuroform Atlas, Stryker, USA), 23 (20%) were braided stents (Leo+ and Leo+ baby, Balt, USA; Lvis Evo, Microvention, USA), and 69 (62%) were FD (Pipeline Embolization Device, Medtronic, USA; Silk Vista and Silk Vista Baby, Balt, USA; Surpass Evolve, Stryker, USA). More use of FD were significantly depicted in group T (*p* < 0.001; 78 vs. 24% in group E and 33% in group C). In group T, stents were preferentially placed in the internal carotid artery (51%, *p* = 0.001), in group E indifferently (*p* = 0.125), in group C in the middle cerebral artery (60%, *p* < 0.001).

### Safety and Efficacy

All the patients' DAPT were switched to oral DAPT maintenance regimen; except for one of the patients in the group C for which antiplatelets were definitely stopped after a vessel rupture. All patients in groups T and E had a relay with Ticagrelor as a maintenance regimen. In group C, 13 patients were switched to Ticagrelor, and 1 to Prasugrel because of respiratory insufficiency.

The overall results concerning safety and efficacy are summarized in Table 4.

**Table 4:** Safety and efficacy evaluation.

**Description (*n*, %)**	**Group T (*****N*** **=** **76)**		**Group E (*****N*** **=** **21)**		**Group C (*****N*** **=** **15)**		***p*-value**
Events	7	9%	4	19%	3	20%	*0.279*
Events adjudicated to be related to DAPT	5	7%	4	19%	2	10%	*0.148*
Symptomatic events	3	4%	3	14%	2	13%	*0.106*
Acute ischemic stroke	1	1%	2	10%	0	0%	
Transient ischemic stroke	0	0%	1	4%	0	0%	
Intracranial hemorrhage	0	0%	0	0%	1	7%	
Others	1	1%	0	0%	1	7%	
Death	1	1%	0	0%	0	0%	
Change in mRS score at 3–6 months	2	3%	2	10%	2	13%	
Asymptomatic events	4	5%	1	4%	1	7%	*1.000*
Silent infarcts	2	2.6%	1	4%	1	7%	
Intracranial hemorrhage	2	2.6%	0	0%	0	0%	
Intra-stent stenosis at 6 months							*0.246*
Number of catheter angiography performed at 6 months	73	96%	19	90%	9	60%	
No significant intra-stent stenosis	63	87%	15	79%	8	89%	
Moderate intra-stent stenosis	9	12%	3	16%	0	0%	
Major intra-stent stenosis	1	1%	1	5%	1	11%	
Aneurysmal occlusion score at 3–4 months							*0.670*
Raymond-Roy 1	52	69%	16	76%	12	80%	
Raymond-Roy 2	15	20%	2	10%	1	7%	
Raymond-Roy 3	8	11%	3	14%	2	13%	

*n: number; %: percentage*.

Eleven events over the 14 recorded were adjudicated (i.e., certainly or likely) to be related to DAPT (10% of events related to DAPT in the total sample), their repartition did not differ between the 3 groups (*p* = 0.432; 7% in group T, 15% in group E, 13% in group C).

All symptomatic events (*N* = 8) were not distributed significantly differently between the 3 groups (*p* = 0.106; 4% of the group T, 14% in group E and 13% in group C) and asymptomatic events were also balanced (*p* = 1.000; 5% in group T, 4% in group E and 7% in group C). Of these 8 symptomatic events, 6 subjects had a change in the mRS score at 3–6 months: 2 in group T (from 5 to 6 and from 0 to 2), 2 in group E (from 1 to 3 and from 0 to 1), 2 in group C (from 0 to 1 and from 3 to 4).

Death occurred in one patient in group T 2 months after the intervention and was related to aspiration pneumonia linked to general condition deterioration caused by a mass effect of a giant carotid aneurysm.

Thrombo-embolic events represented the most encountered events in the sample (8/14). Two acute ischemic strokes were recorded in group E and one in group T. One transient ischemic stroke was noted in group E. Four silent infarcts were found on control MRI (two belonged to group T, one to group E and one to group C). No delayed acute ischemic presentations were reported.

**Figure 2:**
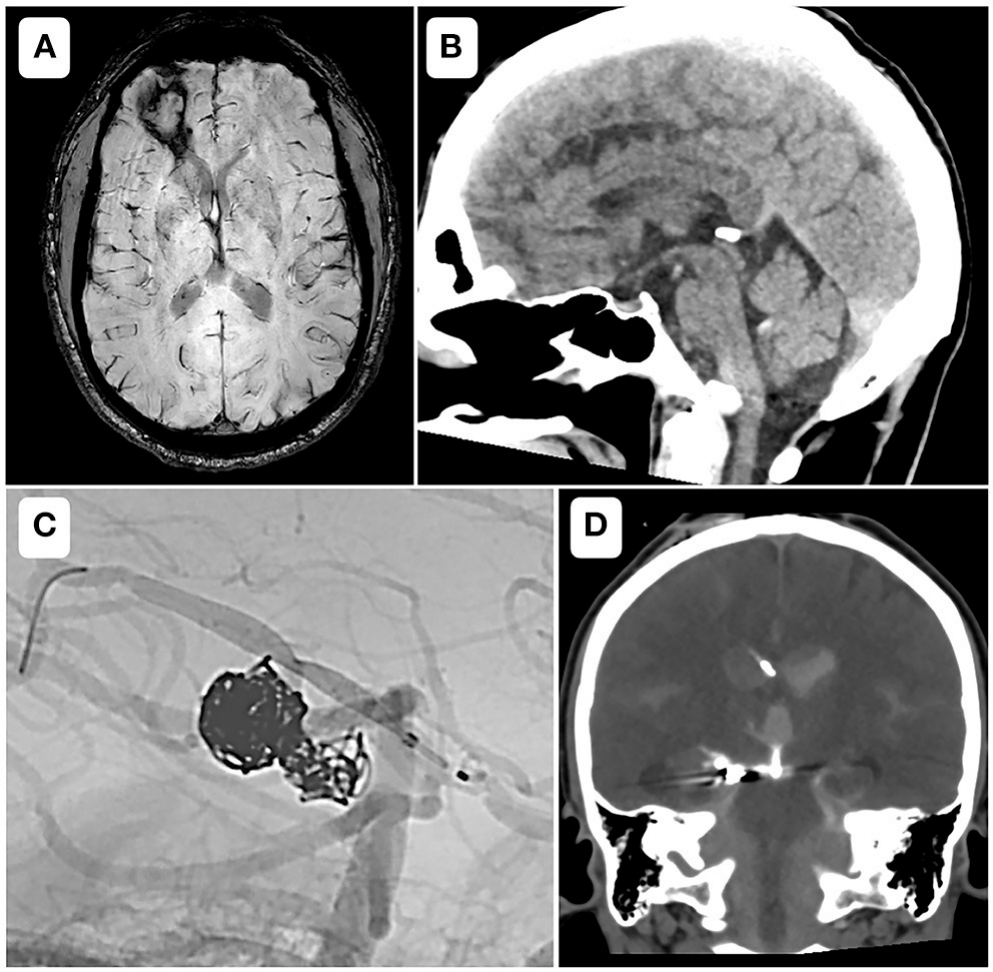
Bleeding events. **(A)** Axial susceptibility weighted imaging, showing a parenchymal bleeding sequelae at 4 months of a stent assisted coiling of the recanalization of a previously ruptured anterior communicating artery aneurysm. The patient belonged to group T and did not present any neurologic deficit. **(B)** Sagittal reformat of a day 1 control CT-scanner, after a placement of a flow diverter to treat an unruptured fusiform aneurysm of the right vertebral artery, showing an isolated intraventricular hemorrhage in the fourth ventricle. The patient belonged to group T and did not present any neurologic deficit. **(C)** Case of a 33-year-old female, modified Rankin Scale 3, with a history of aneurysmal rupture, who underwent a scheduled treatment for an early recanalization of a right posterior cerebral artery aneurysm. After coilling through a microcatheter from the right vertebral artery, a low profile flow diverter was placed through a microcatheter from the right posterior communicating artery. An intra-stent balloon angioplasty provoked a vessel rupture that was jugulated with glue. Heparin was reversed and Cangrelor infusion was stopped. The neurosurgeon was immediately notified and a ventricular derivation was performed within 30 min **(D)**. The patient kept left hemiparesis, with a modified Rankin Scale of 4.

Among three intracranial hemorrhage events, one was symptomatic and occurred in group C (vessel rupture), two were asymptomatic and occurred in group T. These cases are illustrated in the Figure 2.

Two other miscellaneous events were recorded: one pseudo-aneurysm of the superficial femoral artery that required surgical intervention in the group C, and one contrast-induced encephalopathy that occurred in group T.

One hundred and one patients (90%) underwent a follow-up catheter angiography. There was no significant difference in the repartition of intra-stent stenosis between the 3 groups (*p* = 0.246), neither considering the aneurysmal occlusion rates (*p* = 0.670). The occlusion of the target aneurysm was complete at the control imaging (3–6 months) according to Raymond Roy's classification in 69% in group T, 76% in group E and 80% in group C.

### Average Cost

In the group T, all the patients were given previously describe medication, for an average cost of USD 3.54 per patient.

In the group C, two patients required an additional vial of cangrelor due to their weight, for an average cost of USD 457.16 per patient.

In group E, two patients were continued on Eptifibatide for 24 h and one patient for 72 h due to three declared ischemic events, for a mean cost of USD 217.77 per patient.

## Discussion

### Safety and Efficacy

Facing the heterogeneity of practices and studies, the optimal DAPT regimen(s) for interventional neuroradiology cases remain unclear. This study did not depict a significant difference in the repartition of DAPT related events between the three groups of antiplatelet regimens, neither in the rate of all symptomatic (or a symptomatic) events. Thus, our study support the growing literature on the pending demonstration that Cangrelor could be an effective antiplatelet agent for preventing thromboembolic events in situation of stenting, and a safe agent regarding bleeding risk and possibility to reverse its effect rapidly.

The only event that occurred in the group that used Cangrelor that was considered as a thromboembolic complication was a silent infarction on the control MRI. This event did not modify the functional outcome of the patient nor the latter management of DAPT. Cangrelor was found to be useful when decision was made to proceed without DAPT regimen upfront because the need of stenting was not anticipated (6% of the sample), and these results are encouraging in terms of efficacy of the chosen antiplatelet management. Also, the percentage of symptomatic ischemic complications (3%) found in this study is in agreement with the literature: a meta-analysis of symptomatic ischemic complications with the use of a flow diverter by O'Kelly et al.found a rate of 4% (23) and a second meta-analysis with the use of a stent-assisted coiling by Phan et al. found a rate of symptomatic ischemia of 4.5% (1).

The only bleeding event using Cangrelor was symptomatic (Figure 2), and occurred while treating a previously ruptured P2-P3 aneurysm by a low profile FD. The operator decided to inflate the balloon in the stent and experienced an arterial rupture. The heparin was immediately reversed and the infusion of Cangrelor stopped, followed by an occlusion the posterior communicating axis. The neurosurgery team was immediately notified and an external ventricular derivation was performed within 30 min. At 3 months persisted a left hemiparesis, with a mRS score of 4 (for an initial mRS of 3). This case perfectly emphasizes that the rapid offset of Cangrelor allows prompt management during per procedural bleeding complications (i.e., aneurysm rupture, vessel perforation), or need of a surgical intervention (8).

The last event that occurred in patients under Cangrelor was a femoral puncture site false aneurysm, requiring surgical management. The mRS score of this patient was modified at 3 months (from 0 to 1).

The DAPT regimen did not affect aneurysm occlusion at 3–4 months and intra-stent stenosis on arteriography at 6 months.

### Cangrelor Use

A loading dose of Cangrelor of 30 μg/kg and a maintenance dose of 4 μg/kg/min were used in our study. This dosage was derived from the cardiology literature (24). Preliminary studies have investigated Cangrelor at half dose, with a loading dose of 15 μg/kg and a maintenance dose of 2 μg/kg/min (18, 19). These studies did not show an increased ischemic risk. In our center, the choice of Cangrelor was preferred to Ticagrelor when there was a risk of bleeding during the procedure. Further studies are needed to assess the optimal dosage of Cangrelor for endovascular neuro-interventions regarding its safety and efficacy.

The use of Cangrelor requires careful planning with the anesthesia team. The maintenance infusion should be delivered immediately after the loading bolus. We prepared at the same time all syringes and operators had to wait 10 min after the bolus to place the stent and had to paid attention to the injection site patency until the oral relay.

Ticagrelor is the best choice for oral relay because it binds to the P2Y_12_ receptor in a different way than Cangrelor (25). In our study one subject in Group C was given Prasugrel as a relay because of pre-existing respiratory failure, limiting the theoretical use Ticagrelor.

According to the cardiologic literature, Ticagrelor can be given at any time during the infusion of Cangrelor (25). *In vitro* studies have shown interaction of Cangrelor with Prasugrel and Clopidogrel (26). In order to limit these interactions, it is recommended for the relay with Prasugrel to be performed 30 min before the end of the Cangrelor infusion (27). Clopidogrel should only be delivered after the end of the Cangrelor infusion (28). In our practice, we favor a relay with Ticagrelor because of less drug-drug interaction with Cangrelor, and less interpatient response variability.

One should not forget that Cangrelor is still an expensive drug and that cost-effectiveness analysis may be welcome in further studies, similar to that made with Ticagrelor (29).

### Limitation

This was a retrospective, non-randomized, single-center study. Authors acknowledge an obvious selection bias: patients with anticipated procedure-related hemorrhage risk were preferentially treated using Eptifibatide or Cangrelor. Furthermore, the group using a Ticagrelor preparation used more FD without coiling. Similarly, at-risk locations, such as the middle cerebral artery because of the presence of recurrent divisions, were more represented in the groups that received intravenous antiplatelets. These groups (E and C) also included a greater proportion of patients for whom stenting was not initially planned, secondary to coil protrusion or, conversely, to avoid protrusion of a risky coil. Another limitation could be the relative diversity of procedures and devices used, as new medical implant devices with surface modifications are currently being developed and the safety and efficacy profiles of antiplatelets management in these cases should be studied (30, 31).

Our study included 15 patients treated using Cangrelor. To our knowledge, this is the largest current cohort of unruptured aneurysms with the use of Cangrelor (Table 2). Results of safety profile of Cangrelor was compared to two other standardized DAPT regiment in our center, but not compared to a proper control group. Also, different dosing protocols are described in the literature and have not been compared. Further prospective comparative studies with larger cohorts are needed to confirm our results and clarify the best protocols and real comparative safety profiles. Studies of Cangrelor in interventional neuroradiology are still rare. This medication has a rapid onset and offset of action, owing to its short half-life, that fit the demand for neuro-intervention procedures. This preliminary study paves the way for a randomized analysis to confirm its potential for routine use.

## Conclusion

This single-center, retrospective study over 4 years and 3 months compared three DAPT regimens in the context of aneurysm treatment requiring a stent/FD, through the report of their safety and efficacy. From the results, Cangrelor allows for a secure transition to long-term DAPT and secured surgery in cases of unexpected complications. The studies on Cangrelor are still rare with few patients. Randomized controlled studies are warranted to confirm the results of our study.

## Data Availability Statement

The original contributions presented in the study are included in the article/supplementary material, further inquiries can be directed to the corresponding author/s.

## Author Contributions

MN, JG, and EM contributed to conception and design of the study. MC and MA organized the database. JO and MC performed the statistical analysis. MC, JO, and MA wrote the first draft of the manuscript. MC, EM, MA, MN, JG, and JO wrote sections of the manuscript. All authors contributed to manuscript revision, read, and approved the submitted version.

## Conflict of Interest

The authors declare that the research was conducted in the absence of any commercial or financial relationships that could be construed as a potential conflict of interest.

## Publisher's Note

All claims expressed in this article are solely those of the authors and do not necessarily represent those of their affiliated organizations, or those of the publisher, the editors and the reviewers. Any product that may be evaluated in this article, or claim that may be made by its manufacturer, is not guaranteed or endorsed by the publisher.

## Abbreviations

SAC: stent-assisted coiling
FD: flow diverter stents
DAPT: Dual antiplatelet therapy
MRI: Magnetic Resonance Imaging
mRS: modified Rankin Scale.
